# Discrete and Effortful Imagined Movements Do Not Specifically Activate the Autonomic Nervous System

**DOI:** 10.1371/journal.pone.0006769

**Published:** 2009-08-26

**Authors:** Laurent Demougeot, Hervé Normand, Pierre Denise, Charalambos Papaxanthis

**Affiliations:** 1 Université de Bourgogne, UFR STAPS, BP 27877, Dijon, France; 2 Institut National de la Santé et de la Recherche Médicale (INSERM), Unité 887, Motricité et Plasticité, BP 27877, Dijon, France; 3 Université de Caen, Faculté de Médecine, Caen, France; 4 Institut National de la Santé et de la Recherche Médicale (INSERM), ERI27, Caen, France; University of Groningen, Netherlands

## Abstract

**Background:**

The autonomic nervous system (ANS) is activated in parallel with the motor system during cyclical and effortful imagined actions. However, it is not clear whether the ANS is activated during motor imagery of discrete movements and whether this activation is specific to the movement being imagined. Here, we explored these topics by studying the baroreflex control of the cardiovascular system.

**Methodology/Principal Findings:**

Arterial pressure and heart rate were recorded in ten subjects who executed or imagined trunk or leg movements against gravity. Trunk and leg movements result in different physiological reactions (orthostatic hypotension phenomenon) when they are executed. Interestingly, ANS activation significantly, but similarly, increased during imagined trunk and leg movements. Furthermore, we did not observe any physiological modulation during a control mental-arithmetic task or during motor imagery of effortless movements (horizontal wrist displacements).

**Conclusions/Significance:**

We concluded that ANS activation during motor imagery is general and not specific and physiologically prepares the organism for the upcoming effortful action.

## Introduction

Motor imagery is a mental process during which a subject internally simulates an action without any apparent motion of the limbs involved in the physical execution of the same action. Converging evidences from several investigations indicate that motor imagery and motor execution activate similar neural networks and share common motor representations. For instance, many studies have established that neural structures, including the parietal and prefrontal cortices, the supplementary motor area, the premotor and the primary motor cortices, the basal ganglia, the cerebellum, and for some motor tasks the spinal cord, are activated during both overt (executed) and covert (imagined) actions [1]–[3].

The hypothesis that motor imagery and motor execution are functionally linked is also supported by several experiments using the mental chronometry paradigm. These studies have shown that executed and imagined movement durations are very similar for various motor tasks, suggesting that common cognitive processes are shared between overt and covert stages of motor actions [4]–[7].

The activation of the motor system during motor imagery does not result in muscular activation or overt movement execution probably due to an inhibitory mechanism operating downstream to the motor cortex (i.e. by blocking the descending neural commands at the spinal cord or brainstem level). However, this inhibition is not complete, since it is well known that motor imagery involves an autonomic component, which anticipates the metabolic changes produced by the movement itself. Indeed, the findings of several investigations have indicated functional similarities between motor imagery and motor execution at the physiological level [see 8, for a review]. For instance, Decety et al. [9] have reported that both heart rate and total ventilation increase with respect to the resting level during mental simulation of running on a treadmill at several different speeds. This increase was proportional to the simulated running speed and was attributed mainly to central (i.e. preparation for an effort) and less to peripheral (i.e. muscular metabolic activity) mechanisms. This finding indicates that the central processes involved in motor programming can produce, in the absence of muscular contraction, an anticipatory activation of autonomic effectors.

Therefore, the autonomic nervous system (ANS) is activated along with the motor system during preparation for an effortful action, so as to render the action efficient as soon as muscles begin to contract. However, in the studies cited above, motor tasks were highly automatic, cyclical and required great effort from the subjects. The question remains whether the activation of the autonomic system persists during discrete movements which do not require a repeated and major effort. Furthermore, up to now it has not been established whether the activation of the ANS during motor imagery is specific to the action being imagined. In order to explore these topics, we make use of the baroreflex control of the cardiovascular system. When a subject straightens his trunk from the supine position, the amount of blood volume abruptly decreases in the head and the trunk and increases in the legs. As a consequence, arterial pressure (AP) decreases significantly (orthostatic hypotension) and, in order to keep the initial blood volume in the trunk, the physiological reaction of the organism consists in increasing the heart rate (HR) and peripheral vascular resistance until the reestablishment of AP. Is this physiological process also present when a subject, instead of executing, imagines himself straightening his trunk? On the other hand, when a subject from the supine position moves his legs upwards the amount of blood decreases in legs and increases in the trunk. This action causes a small increase in the HR (due to the effort made to move the legs against gravity) and no or a tiny increase in the AP. Does the same physiological reaction take place when a subject, instead of executing, imagines himself moving his legs upwards? We assume that, if the activation of ANS during motor imagery is specific, then one could anticipate greater increase in HR and AP during imagined trunk than imagined leg movements. On the other hand, if ANS activation is general and not specific (i.e. anticipation of an upcoming action) then the HR and AP should be similarly modified in the case of trunk and leg imagined movements.

## Materials and Methods

### Ethical statement

The regional ethics committee of Burgundy (C.E.R) approved the experimental protocol which was carried out in agreement with legal requirements and international norms (Declaration of Helsinki, 1964).

### Participants

Ten healthy male participants, aged between 23 and 31 years (mean age = 26.3±2.1 years), voluntarily participated in the present experiment (informed consent was signed). Participants were screened for the following exclusion criteria: vascular, cardiorespiratory, neurological or motor diseases, regular taking of drugs or medication. Their motor imagery ability was assessed using the French version of the Movement Imagery Questionnaire [MIQr; 10]. The MIQr measures the difficulty of forming visual and kinaesthetic images of movements on a 7-point scale (1: very difficult; 7: very easy) in 8 items (maximum score: 56; visual modality: 28; kinaesthetic modality: 28). All participants were judged good imagers, as they reported scores higher than 40 (mean = 43.4±2.4; visual modality = 21.6±1.7; kinaesthetic modality = 21.8±0.9). The participants received complete information about the experimental procedures before the experiment, but none of them was informed about the specific hypothesis of the study.

### Motor tasks and Experimental procedure

**Figure 1:**
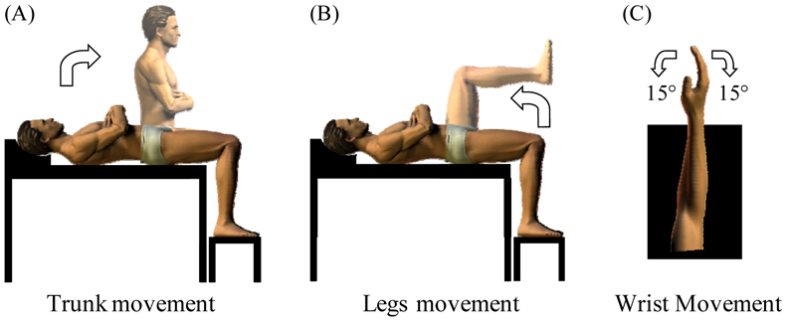
Motor tasks. (A) Trunk and (B) leg movements (right-side view). Participants executed and imagined *trunk* and *leg* movements (amplitude 90°) from the resting position (lying down). (C) Wrist movements (top view). Participants executed and imagined *wrist* movements (amplitude 30°: 15° leftwards and 15° rightwards from the ulnar axis) in the horizontal plane.

The experiment took place in a quiet room (5×5 m) which was temperature regulated (∼22°C) and illuminated with homogeneous white light. Participants were lying comfortably on a physiotherapist table in the supine position with their arms crossed over the trunk. From that initial position, the participants were asked to execute or to mentally simulate (imagine) two motor tasks. The first one (Fig. 1A) consisted of elevating the trunk to the vertical position (*movement* phase) and remaining in that position (*posture* phase, hip angle ∼90°) for 5 s. The second task (Fig. 1B) consisted of elevating both legs to the vertical position (*movement* phase) and remaining in that position (*posture* phase, hip and knee angles ∼90°) for 5 s. Participants were requested to execute and to mentally simulate the motor tasks at a natural, self selected, speed and with their eyes open. We specified that they must feel themselves performing the task (motor or internal imagery) rather than just visualizing themselves (visual or external imagery). All the tasks were carry out in end expiratory, open glottis apnea in order to avoid variation in autonomic activity due to breathing.

The executed and the imagined trials lasted from ∼45 s to ∼ 90 s and were proceeded as follows: (1) the participants remained relaxed on the table for at least 30 s (until the recorded physiological parameters reached a stable level); (2) after this period and at the end of a breath cycle (i.e. end of expiratory phase), the participants were requested by the experimenter to stop breathing; (3) after 5 s, a vocal “go” signal indicated to the participants to start the execution or the mental simulation of the movement; (4) consequently, the participants executed or mentally simulated the *movement* phase (they indicated the beginning and the end of this phase by pressing a handheld push-button) and the *posture* phase (5 s, until the vocal “stop” signal by the experimenter); (5) after the trial completion, participants returned to their initial position (executed trials only).

We compared the duration of the *movement* phase between the executed (2.50±0.20 s) and the imagined (2.55±0.29 s) trials and we found no significant difference (paired t-tests, t = −0.38, *p*>.05). None of the participants expressed any difficulty with apnea. The order of performance of the experimental conditions (*trunk*, *legs*, *executed*, *imagined*) was counterbalanced among the participants. Each participant accomplished 12 trials in each experimental condition (i.e. a total of 48 trials per participant).

### Recording of Physiological parameters (AP, HR)

Non-invasive radial blood pressure was measured by means of a Colin CBM-7000 monitor (Colin Corp., Komaki City, Japan), using the arterial applanation tonometry technique [11] to record the pulse wave of the radial artery. During the experiments the sensor was replaced when the position indicators went beyond the recommended range. Therefore, care was taken so that the tonogram displayed a single peak, that the signal strength (an indicator of the quality of the signal) was over 60% and that the hold-down pressure (mean pressure applied against the skin) was between 40 and 120 mmHg. The device was calibrated using a standard automated oscillometric blood pressure measurement obtained from a brachial cuff. The calibration procedure of the Colin monitor was initiated manually at the beginning of each serie and each time the tonometric sensor was repositioned. The first measurement was taken at least 10 minutes after the Colin CM-7000 was positioned. Heart rate was recorded via a standard ECG monitor (Gould) using sternal manubrium to C5 derivation. Signals were digitized online with a 1 kHz sampling frequency and a 12-bit resolution. Data were saved on a computer and analyzed off-line. Artefacts were manually removed during examination of raw data plots. Systolic and diastolic blood pressures were determined using a peak detector. Mean AP was calculated from the averaged areas under the pressure waveform (Notocord-Hem 3.5, Croissy sur Seine, France).

### Recording of Electromyographic activity (EMG)

In order to verify that motor imagery was purely mental and that physiological parameters (i.e. AP and HR) were not altered by peripheral factors (i.e. weak muscle contraction), we controlled the level of muscle activation during imagined movements. EMG signals were recorded from the right Sternocleidomastoideus (head flexor and rotator), the right Rectus Abdominis (trunk flexor) and the right Rectus Femoris (hip flexor) muscles. Two silver-chloride electrodes were placed on the muscle belly (the skin previously shaved and cleaned) with an inter-electrode distance (centre to centre) of 2 cm. EMG signals were recorded using a Biopac (Santa Barbara, USA), sampled at 2000 Hz, band pass filtered (30–300 Hz).

### Data and statistical analysis

**Figure 2:**
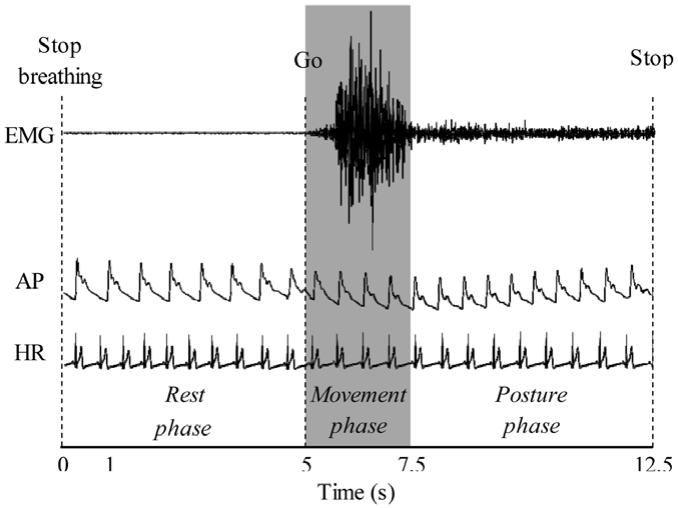
Data recording. Typical data of EMG activity, Arterial Pressure (AP) and Heart Rate (HR) are illustrated for the *rest*, the *movement* and the *posture* phases during an executed movement.

AP, HR and EMG signals during executed and imagined trials were analyzed from the moment that participants stopped breathing until the end of the trial (Fig. 2). We decomposed this period into three successive phases: the *rest* phase (5 s; from the verbal “stop breathing” signal until the verbal “go” signal; the experimenter marked these instants on the EMG patterns by means of a switch), the *movement* phase (the participants indicated the beginning and the end of this phase by pressing a handheld push-button; two markers indicated these instants on the EMG pattern) and the *posture* phase (from the end of the *movement* phase until the end of the trial).

We included several steps in our statistical analysis of AP and HR. First, for each participant, we calculated the average values of AP and HR for the *trunk* and *leg* conditions during the *rest*, *movement* and *posture* phases. All the variables were normally distributed as neither the Kolmogorov-Smirnov & Lilliefors test nor the Shapiro-Wilk test were significant (*p*>.05). Then, we analysed the AP and HR of the two motor tasks by performing a repeated-measures analysis of variance (ANOVA) with *body-segment* (*trunk* and *legs*) and *action-type* (*rest, movement, posture*) as within-subject factors. This analysis was performed for the *executed* and the *imagined* conditions separately. For each ANOVA, we reported the epsilon (*ε*) values (*Geisser-Greenhouse*) and the *post-hoc* differences were evaluated by means of *Scheffé* tests (significance: *p*<.05).

Custom off-line processing (MatLab, The MathWorks Inc., Natick MA) was used in order to quantify muscle activation. We computed the Root Mean Square (RMS) values of EMG signals:
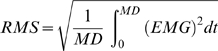
where MD is movement duration.

We first analyzed the EMG signals of executed movements. The onset of muscle activity was defined as two standard deviations above the baseline activity (i.e. activity during the *rest* phase) lasting for more than 20 ms. We observed that muscle activation increased immediately after the participants pressed the push-button (indicating the beginning of the *movement* phase) and continued until the end of the movement.

The RMS values of the *rest* phase (RMS_R_) were used to determine the baseline level (muscles totally relaxed), while those of the *movement* (RMS_M_) and *posture* (RMS_P_) phases were used to assess any increase in muscle activation during internal movement simulation. For each participant, we calculated the average RMS values of the *rest*, *movement* and *posture* phases. These variables revealed a normal distribution (Kolmogorov-Smirnov & Lilliefors test and Shapiro-Wilk tests were not significant; *p*>.05) and therefore we compared, by means of paired *t-tests*, the RMS values of the *rest* phase with those of the *movement* or the *posture* phase. This analysis was performed for each muscle separately in both the *trunk* and *legs* conditions. We completed our statistical analysis by examining the same statistical differences at the individual level. Comparisons (paired *t-tests*) of the RMS values of the *rest* phase with those of the *movement* or the *posture* phase were applied for each participant.

## Results

### Arterial Pressure (AP) and heart rate (HR) during executed movements

**Figure 3:**
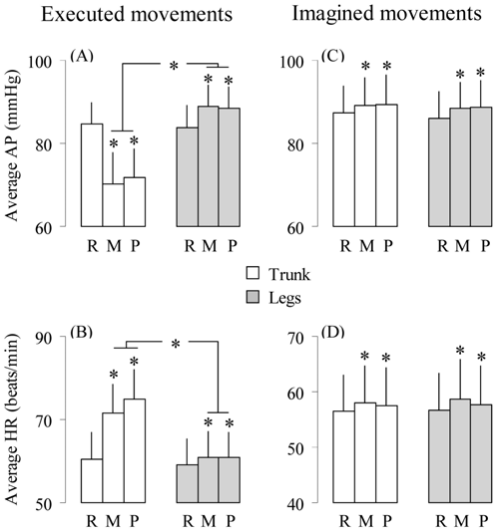
Arterial pressure (AP) and heart rate (HR). Average values (±SD) of AP and HR during the *rest* (R), the *movement* (M) and the *posture* (P) phases are illustrated for both *trunk* (white bars) and *leg* movements (grey bars). (A–B) *executed* movements. (C–D) *imagined* movements. Stars (*) indicate significant differences (*p*<.05).

Fig. 3A and 3B respectively show the average values of AP and HR for the *executed* condition. For AP, ANOVA revealed a significant main effect for *body-segment* (F_1,9_ = 12.46; *p = *.016; *ε = 1*). Precisely, AP was greater for the *legs* (on average 75.5 mmHg) than the *trunk* (on average 87.1 mmHg) condition. There was also an interaction effect between *body-segment* and *action-type* (F_2,18_ = 23.02, *p*<.001; *ε = 0.94*). The *post-hoc* analysis showed that AP values between the *trunk* and *legs* conditions were similar during the *rest* phase (*p*>.05) but significantly different during the *movement* (*p*<.001) and the *posture* (*p*<.001) phases. Furthermore, in the *trunk* condition, AP significantly decreased between the *rest* and the *movement* phases (−19.7%; *p*<.001) or between the *rest* and the *posture* phases (−16.8%; *p*<.001), while it remained similar between the *movement* and the *posture* phases (*p*>.05). On the contrary, in the *legs* condition, AP slightly, but significantly, increased between the *rest* and the *movement* phases (7.1%; *p*<.05) or between the *rest* and the *posture* phases (6.6%; *p*<.05), while it remained similar between the *movement* and the *posture* phases (*p*>.05).

In terms of HR, ANOVA revealed a significant main effect for *body-segment* (F_1,9_ = 128.06; *p*<.001; *ε = 1*; on average: 69 beats.min^−1^ for the *trunk* and 60 beats.min^−1^ for the *legs*). There was also an interaction effect between *body-segment* and *action-type* (F_2,18_ = 81.71, *p*<.001; *ε = 1*). The *post hoc* analysis showed that HR values between *trunk* and *legs* conditions were similar during the *rest* phase (*p*>.05) but significantly different during the *movement* (*p*<.001) and the *posture* (*p*<.001) phases. Furthermore, for the *trunk* condition, HR significantly increased between the *rest* and the *movement* phase (20.3%; *p*<.001), between the *rest* and the *posture* phases (24.4%; *p*<.001) and between the *movement* and the *posture* phases (4.1%; *p*<.001). Similarly, for the *legs* condition, AP slightly, but significantly, increased between the *rest* and the *movement* phases (3.8%; *p*<.05) or between the *rest* and the *posture* phases (4.1%; *p*<.05), while it remained similar between the *movement* and the *posture* phases (*p*>.05).

### Arterial Pressure (AP) and heart rate (HR) during imagined movements

The average values of AP and HR for the *imagined* movements are respectively depicted in the Fig. 3C and D. For AP, ANOVA revealed a significant main effect for the *action-type* (F_2,18_ = 28.43; *p*<.001; *ε = 1*). Precisely, AP increased between the *rest* and the *movement* phases (2.8%; *p*<.001) and between the *rest* and the *posture* phases (2.9%; *p*<.001), but remained similar between the *movement* and the *posture* phases (*p*>.05).

For HR, ANOVA also revealed a significant main effect of the *action-type* (F_2,18_ = 10.9; *p* = 0.001; *ε = 1*). Specifically, HR increased between the *rest* and the *movement* phases (3.1%; *p*<.001) and between the *rest* and the *posture* phases (2.7%; *p*<.001), but remained similar between the *movement* and the *posture* phases (*p*>.05). ANOVA did not reveal a main effect of *body-segment* or an interaction between *body-segment* and *action-type* (for both *p*>.05).

**Figure 4:**
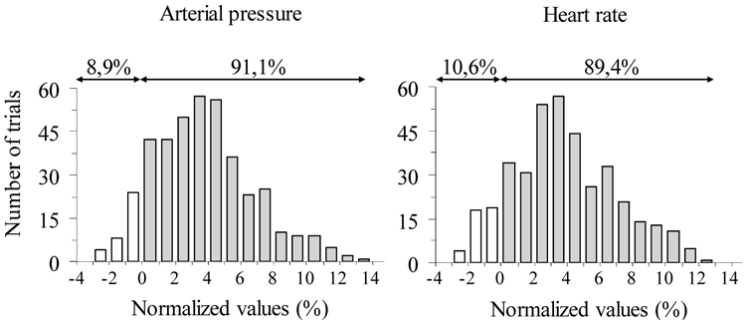
Normalized values of Arterial Pressure (AP) and Heart Rate (HR). The distribution of normalized values of AP (AP = MP_AP_/R_AP_ ×100−100) and HR (HR = MP_HR_/R_HR_ ×100−100) are shown for the imagined *trunk* and *leg* movements. Arrows represent the proportion of positive (grey bars) and negative (white bars) normalized values. MP_AP_: average AP during the *movement* and the *posture* phases; R_AP:_ average AP during the *rest* phase; MP_HR_: average HR during the *movement* and the *posture* phases: R_HR_: average HR during the *rest* phase.

The above analysis revealed a significant increase in AP and HR during imagined *trunk* and *legs* movements. In order to further analyse these findings, we calculated the following ratios trial-by-trial: (1) (MP_AP_/R_AP_ ×100) −100; where MP_AP_ is the average AP during the *movement* and the *posture* phases and R_AP_ is the average AP during the *rest* phase; (2) (MP_HR_/R_HR_ ×100) −100; where average MP_HR_ is the average HR during the *movement* and the *posture* phases and R_HR_ is the average HR during the *rest* phase. Fig. 4 shows the distribution of these two ratios (n = 432 for each ratio) for the imagined movements. Positive and negative normalized values indicate respectively an increase and a decrease in AP or HR with respect to the baseline. It is worth noting, that in more than 89.4% of the trials, AP and HR increased during the imagined trials, thus suggesting that the activation of ANS is a consistent phenomenon, observed in all the participants.

### EMG analysis during imagined movements

**Figure 5:**
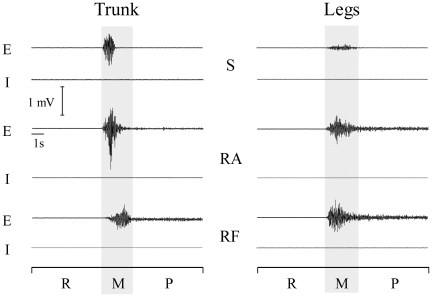
EMG patterns. Typical EMG patterns from one participant are depicted for executed (E) and imagined (I) *trunk* and *legs* movements during the *rest* (R), the *movement* (M) and the *posture* (P) phases. S: Sternocleidomastoideus, RA: Rectus Abdominis, RF: Rectus Femoris.

The analysis of EMG patterns permitted us to discard any effect of peripheral factors (i.e. weak muscle contraction) upon AP and HR enhancement during motor imagery. Fig. 5 qualitatively illustrates typical EMG activities in trunk and legs muscles during one executed and one imagined trial. It is noticeable that, sternocleidomastoideus, Rectus Abdominis and Rectus Femoris muscles are active during the *movement* phase (concentric contraction). Rectus Abdominis and Rectus Femoris muscles remained active during the *posture* phase (isometric contraction) while sternocleidomastoideus muscle was relatively silent. Interestingly, during motor imagery, these muscles remained silent during both the *movement* and the *posture* phases.

In terms of *trunk* movements, the statistical analysis (paired *t-test*), performed for each muscle separately, revealed no significant difference between the *movement* and the *rest* phases or between the *posture* and the *rest* phases (t<−1; *p*>.07, for all comparisons). Similarly, no supplementary EMG activity was revealed for any muscle during the *legs* movements (t<−1.5, *p*>.13, for all comparisons). These statistical results indicate that trunk and leg muscles remained inactive during imagined movements. The mean RMS values of the Sternocleidomastoideus muscle were 0.140±0.038 mV, 0.141±0.041 mV and 0.143±0.044 mV respectively for the *rest*, *movement* and *posture* phases. The same values for the Rectus Abdominis muscle were respectively 0.070±0.087 mV, 0.071±0.129 mV and 0.072±0.139 mV; while for the Rectus Femoris they were 0.086±0.037 mV, 0.081±0.012 mV and 0.087±0.037 mV.

The same statistical analysis on the EMG data was performed for each participant separately. The comparisons revealed no significant difference (t<−1.7, *p*>.05 for all comparisons), thus suggesting that the absence of EMG activity during motor imagery was a consistent phenomenon, observed in all the participants.

### Control conditions

After completion of the experiments, the participants performed two additional-control tasks. The first was a *cognitive* task (i.e mental arithmetic). Our purpose was to explore whether the mobilization of cognitive resources activates the ANS and to compare the degree of this activation with that observed during imagined movements of the trunk and the legs. We considered that the activation of the ANS during the cognitive task, to a comparable level with that recorded during the imagined motor tasks, would indicate that the activation of the ANS during imagined movements could be due to the mobilisation of cognitive resources rather than to the action simulation *per se*. In the opposite case, i.e. no activation of the ANS during the cognitive task, one could suppose that the increase in HR and AP during imagined actions was directly linked to the internal simulation of the trunk or the leg movements. The participants were lying comfortably on a physiotherapist table and were required to subtract 4 and to add 3 starting from the number 50 up to the number 40 (i.e. 50, 46, 49… 40). They had to mentally calculate as accurately and as quickly as possible. The mean duration of this task was 10.4±1.8 s.

We also verified whether the increase in physiological parameters during imagined movements was inherent to the motor imagery process *per se*, rather than to the internal simulation of a specific motor action. In particular, one might suspect that every imagined movement (effortless or not) would activate the ANS. Therefore, our goal in the second control task was to observe whether the internal simulation of an effortless movement (i.e. a movement whose physical execution does not stimulate the ANS above the baseline level) activates the ANS and to compare the degree of this activation with that observed during imagined movements of the trunk and the legs. For that purpose, the participants executed or imagined cyclical wrist movements at a natural, self selected, speed. We considered that an increase in physiological parameters during the imagined wrist movements, comparable to that observed during imagined trunk or leg movements, would indicate that the activation of the ANS during imagined movements was intrinsic to the motor imagery process rather to the internal simulation of a physically demanding movement, such as a trunk or a leg displacement. In the opposite case, one could suppose that the increase in HR during imagined actions was specific and related to the dynamic features of the movement being imagined. Participants were lying comfortably on a physiotherapist table; they held their right arm fully extended (upper arm and forearm were supported by a table) and they maintained their right wrist in a semipronated position (Fig. 1C). From that initial position they executed or imagined wrist displacements of 30° (15° leftward and 15° rightward from the ulnar axis in the horizontal plane) for 10 s.

**Figure 6:**
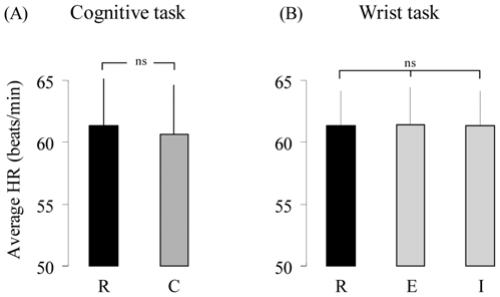
Heart rate (HR) during control conditions. (A) Average values (±SD) of HR for the *cognitive* (C) task and the *rest* (R) condition. (B) Average values (±SD) of HR for the *executed* (E) and *imagined* (I) wrist movements and for the *rest* (R) condition.

In both control tasks, the participants remained relaxed on the table for at least 30 s (until physiological parameters were stable); after this period and at the end of a breath cycle (i.e. end of breathing out), they were requested by the experimenter to stop breathing; after 5 s, a vocal “go” signal indicated the beginning of the control task. In the two control conditions, we measured only HR since a preliminary study showed qualitatively similar results for AP and HR. For each participant, the average values of HR were calculated for the *cognitive* task during the *rest* and the *mental calculation* phases. Similarly, the average values of HR were calculated for the *wrist* task during the *rest*, the *imagined* and the *executed* phases. Then, we compared the average values of HR as follow: 1) between the two phases of the *cognitive* task (i.e. *rest* and *cognitive*) by means of paired t-tests; 2) between the three phases of the *wrist* condition (i.e. *rest*, *imagined* and *executed*) by means of an ANOVA (repeated measures). Fig. 6A shows the average values of HR for the *rest* and the *mental calculation* phases. It clearly appears that HR did not increase when participants performed an arithmetic task (paired *t-test*; t = 0.94; *p*>.05). The average values of HR for the *rest*, *executed* and i*magined* phases during *wrist* movements are depicted in the Fig. 6B. It is noticeable that HR was similar among the three phases. This observation was confirmed by ANOVA which revealed no significant effect (F_2,18_ = 0.03; *p*>.05; *ε = 0.89*).

## Discussion

The aim of the present study was to examine whether the internal simulation of effortful discrete movements activates the ANS and whether this activation specifically parallels the physiological changes that occur in the organism during the physical execution of the same movements. The main findings of our study indicate that physiological parameters (i.e. AP and HR) significantly increased during the mental simulation of trunk or leg movements against gravity. Interestingly, such physiological modulation was not observed during a mental-arithmetic task or during the mental simulation of an effortless movement (i.e. horizontal displacements of the wrist). Assuming that motor imagery shares similar neural process with motor execution [1]–[3], our findings indicate that the activation of the ANS through central mechanisms prepares the organism, from a physiological point of view, for the upcoming effortful movements.

However, this anticipation is general and not specific. We found, due to the orthostatic hypotension phenomenon, that HR was significantly greater during the execution of trunk than leg movements. On the contrary, HR increase was of similar amount for the imagined trunk and leg movements. In addition, executed trunk movements induced a decrease in AP because of central blood volume displacement toward the legs, whereas legs movements induced an increase in AP because of the opposite central blood volume displacement. If such a specific anticipatory mechanism existed during imagined actions, one would expect a differential effect of trunk and leg movements on AP, which was not the case. This is a novel finding and denotes that central mechanisms during action planning, whatever if this action is finally executed or not (as it is the case during imagined movements), anticipate the physiological changes of an upcoming motor task, while peripheral mechanisms (present only during executed movements) more precisely respond to the specific physiological requirements during the motor task.

### Autonomic Nervous System activation during motor imagery

Modifications in cardiovascular parameters may be elicited by peripheral factors (for example, from a rise in the activity of chemosensitive afferents within working muscles [12], [13] or from the activation of autonomic reflexes, such as the baroreflexes), and/or by centrally generated neural commands during motor programming used to mobilize the musculoskeletal system [14], [15].

In our study, the absence of supplementary EMG activity during motor imagery with respect to the baseline argues in favour of the central hypothesis. Autonomic activation during imagined actions pertains to the more general phenomenon of motor planning [16]. The activation of the ANS during mentally simulated trunk and leg movements, reported in this study, corroborates the results of previous investigations which suggest that autonomic function, normally associated with muscular activity, can also be triggered during imagined actions [for a review, see 8]. Several studies have shown that motor imagery activates the cardiovascular and respiratory system during prolonged exercises [9], [16], [17]. For instance, Decety et al. [9] have reported that mental simulation of running on a treadmill causes an increase in heart and respiratory rate, proportional to the simulated running speed. Similarly, measurements of cardiac and respiratory activity during physical or mental locomotion at increasing speeds revealed a co-variation of heart rate and pulmonary ventilation with the degree of imagined effort [18]. In addition, Paccalin and Jeannerod [19] have reported an increase in breathing rate during the observation of effortful actions and Papadelis et al. [20] have shown that heart and respiratory rates significantly increased during imagery training in an electronic flight simulation program.

Here, we have demonstrated that the ANS is also activated during mental simulation of discrete movements. This finding is original and indicates that during motor planning of effortful actions, such trunk and leg movements against gravity in our experiment, the Central Nervous System (CNS) both specifies neural commands and physiologically prepares the organism for the upcoming movement. This may allow the muscular and cardiovascular systems to be efficient as soon as the movement starts. This premise is further supported by the finding that the ANS is not triggered during the mental simulation of effortless movements, i.e. wrist displacements. However, it is important to note that the activation of the ANS by central mechanisms, elicited during motor imagery, does not exactly parallel its activation by both central and peripheral factors, elicited during physical execution. Indeed, our findings showed that increase in HR was similar during imagined trunk and leg movements, while it was significantly greater during the physical execution of trunk as compared to leg movements, due to the orthostatic hypotension phenomenon. Also, if central activation had to anticipate physiological reactions due to changes in body position, we would expect AP to decrease during imagined leg movement in order to counterbalance the increase in AP due to the increase in central blood. It is therefore clear that general and not specific central mechanisms during motor planning anticipate the physiological demands of a motor task, while peripheral mechanisms more precisely respond to the physiological changes during a physical effort.

### Computational framework for motor imagery and autonomic nervous system

From a functional point of view, computational models of motor control, which posit the existence of internal models in the brain [21], [22], could be useful in interpreting the ANS activation by central mechanisms. During physical execution, the inverse internal model prepares and generates the appropriate neural commands necessary to drive the trunk or the legs from the initial to the final position. In parallel, an efferent copy of these motor commands is available to the forward internal model, which predicts, before movement initiation, the future states of the trunk or the legs and thus anticipates the sensory consequences of the movement. We consider that the efferent copy of the motor command during action planning has a double function: first, it contributes to motor prediction (internal forward model); second, it anticipates the metabolic changes (ANS activation) produced by the upcoming movement. Detailed analysis of cardiac and respiratory rate changes at the onset of voluntary effort has shown that these changes tend to anticipate muscular activity [15]. During motor imagery, the inverse internal model prepares the appropriate motor command but no overt movement occurs because the motor command is inhibited, probably at the spinal cord or brainstem level. However, an efferent copy of the motor command is still available for the CNS for sensorimotor prediction and physiological preparation of the organism (ANS activation). Due to the efferent motor commands, available before movement initiation and present in both physical execution and mental simulation, the CNS activates the ANS.

From an anatomical point of view, it seems that common neural structures participate in motor prediction, execution and regulation of autonomic activity. This may offer a neurophysiological basis for interpreting our results. It is already well established that mental simulation of movement and its actual execution share a common neural substrate [for a revue, see 1]. Precisely, the parietal and prefrontal cortices, the supplementary motor area, the premotor and primary motor cortices, the basal ganglia and the cerebellum, are activated during both executed and imagined actions. Notably, the cerebellum and the posterior parietal cortex are neural structures which are particularly involved in sensorimotor prediction and mental movement simulation [1], [23]. Recently, it has been found that the ANS activity is partially interlocked with the activity of motor brain regions and that the sympathetic activity to very different organs is mainly predicted by activity in motor brain regions, both pyramidal and extrapyramidal [24]. The activity of the sympathetic nervous system has been directly correlated to activity in the primary and supplementary motor cortices and it has been inversely correlated to the activity in the extrapyramidal motor system i.e. the caudate nucleus. Activity of the cardiac vagal system has been positively correlated to activation of the extrapyramidal motor system.

### Potential interest for rehabilitation

Mental practice improves motor performance [25]–[27] and enhances muscular force [28], [29]. Accordingly, several studies have proposed motor imagery as a complementary method in stroke rehabilitation [for a review, see 30]. Here, our results raise the question of the use of mental practice as a tool for heart failure rehabilitation; notably, in patients who are incapable of performing repetitive movements or lifting loads at the beginning of their rehabilitation. Practised in conjunction with classical methods, motor imagery could decrease the duration of heart failure rehabilitation. Moreover, ANS activation during imagined movements may provide useful indications to physical therapists for the level of effort that patients develop during rehabilitation by means of mental training. This can help to optimize mental training and to minimize discomfort or fatigue for the patients.
